# Conformal radiotherapy and molecular imaging: complementary technologies in cancer therapy

**DOI:** 10.2349/biij.2.1.e2

**Published:** 2006-01-01

**Authors:** MP Mac Manus

**Affiliations:** Department of Radiation Oncology, Peter MacCallum Cancer Institute, East Melbourne, Australia

This issue of the journal highlights some of the significant advances that have taken place in radiotherapy in recent years. The use of ionising radiation has a venerable history in cancer treatment. The first recorded radiobiology ‘experiment’ occurred in 1898 when Henri Becquerel developed a skin reaction to a vial of radium kept in his shirt pocket. Skin cancers were successfully treated in Stockholm as early as 1899. Throughout the 20th century, rapid technological advances led to the development of orthovoltage x-ray therapy machines, and then in rapid succession, the linear accelerator or ‘linac’ and telecobalt apparatus. The development of computer technology for treatment planning and delivery late in the 20th century and more recently the availability of advanced linear accelerators with multileaf collimators, capable of independent movement, has transformed the capability of radiotherapy to accurately target localised cancers. Radiotherapy has been transformed from a discipline that was in danger of stagnation and was becoming marginalised by the rapid advances in systemic therapy, to a dynamic high-technology therapeutic modality at the centre of combined modality therapy for a majority of the most common cancers. A significant factor in this resurgence of radiotherapy is the recently enhanced ability to precisely deliver therapy to the sites of gross disease and to simultaneously reduce irradiation of healthy normal tissues. This has the potential to minimise toxicity while maximising the chance for disease control.

For patients with potentially curable locally or locoregionally advanced disease, conformal three dimensional treatment planning is now routine. The availability of complex dosimetric information allows the routine use of dose volume histogram analysis to determine the dose delivered to precise volumes of normal tissues. This information is extremely valuable in optimising treatment planning to give the safest possible dose distribution to normal tissues while adequately and uniformly delivering dose to tumour volumes. For the most complex planning situations, the routine use of intensity modulated radiotherapy (IMRT) facilitates the delivery of therapy to irregular three dimensional shapes, often with concave regions; an especially remarkable achievement given that photons travel in straight lines! IMRT has already shown clinical utility in prostate cancer, allowing very high doses to be delivered with acceptable toxicity and with emerging evidence of superior disease control [1]. Another outstanding clinical example is the use of parotid sparing IMRT to obtain tumour control without unacceptable toxicity in head and neck cancers [2], especially carcinomas of the nasopharynx [3]. A chronically dry mouth has historically been one of the most distressing toxicities of head and neck radiotherapy [4]. These complex techniques are time consuming, requiring laborious contouring of tumour and normal tissues on planning CT scans and they demand teamwork from radiation oncologists, physicists, dosimetrists and radiation therapists. Further work is required to prove that the additional complexity and expense is worthwhile in a range of common clinical scenarios. In many clinical situations, IMRT might actually represent an unduly costly treatment option.

As our capacity to accurately deliver ionising radiation in cancer therapy has increased, it has become very clear that an accurate assessment of the distribution of tumour in 3-dimensional (and more recently, four dimensional) space is essential. The basis for conformal radiotherapy planning has long been the CT scan. Because of the information on electron density contained in the CT dataset, there is no better medium for determining dose distribution in three dimensions. However, at many disease sites, the CT scan has serious limitations for delineating the true tumour extent. One of the best characterised disease entities where CT is deficient is non-small cell lung cancer (NSCLC). Surgical series have shown that CT scanning is quite poor at determining the true status of mediastinal lymph nodes, a crucially important parameter when determining the target volumes of the thorax to irradiate in a patient with unresectable NSCLC. Another example is lymphoma where CT scanning is very poor at showing disease in a non-enlarged spleen or at other extranodal sites such as bowel or salivary gland and cannot detect disease in non-enlarged lymph nodes. These deficiencies in CT imaging are a major problem for planning radiotherapy, when imaging must be relied upon to determine the gross tumour volumes. Another crucial area where imaging can help is in patient selection for aggressive therapy. CT scanning and other conventional imaging often fails to detect gross distant metastasis and many patients have historically received futile radical radiotherapy when they had incurable disease at the outset.

It is fortuitous that one of the major recent advances in the management of cancer has been the rapid progress in molecular imaging with positron emission tomography (PET) and more recently with integrated PET/CT scanners that simultaneously acquire structural and metabolic information. PET scanning provides complementary staging information to CT and can greatly increase the accuracy of disease assessment in a range of common cancers. The most successful PET radiopharmaceutical has been 18F-fluorodeoxyglucose (FDG), a glucose analogue that is selectively taken up by and trapped in tumour cells. FDG-PET has proven to be of particular value in improving the quality of staging, not only in a wide range of epithelial cancers, including lung, head and neck, cervix, bowel and oesophageal cancers, but also in malignant melanoma, soft tissue sarcomas and in lymphomas. A meta-analysis has proven the superiority of PET over CT in the staging of the mediastinum in NSCLC [5]. The increasing use of PET and especially PET/CT for staging cancer and for determining the spatial distribution of local and locoregional disease has shown us that, in the past, our assessments of cancer patients with conventional imaging have often been inadequate. Even if patients selected for radical radiotherapy really do have potentially curable locoregionally-confined disease, without PET, many of them would have had radiotherapy plans that failed to treat their disease adequately because of geographic miss.

Data from our prospective studies at the Peter MacCallum Cancer Centre [6] and other series show that PET can detect disease too advanced for aggressive therapy in about one third of candidates for radical radiotherapy with NSCLC. These patients would be unable to benefit from an intensive and toxic local therapy and can be spared from futile radical chemoradiation because of PET. Simply by using PET to exclude patients with a poor prognosis, much higher survival can be observed in a series of patients treated with radiotherapy, primarily as a result of better patient selection [7]. Treatment planning studies, including those from our own centre and from the University of Washington [8] suggest that, without PET, a quarter or more of this patient population would have a geographic miss of some gross tumour. Therefore, without PET scanning, dose escalation using our new radiotherapy capabilities would be futile in many cases of NSCLC. Although lung cancer is the malignancy for which the utility of PET in radiotherapy planning is best established, evidence is accumulating to suggest that it may be useful in other cancers such as oesophageal, and head and neck cancers [9].

It would be a mistake however, to emphasise the technical advances in radiotherapy and imaging in isolation. Radiotherapy by itself, no matter how technically advanced, can never be a curative therapy in its own right for most patients with malignant disease. Rapid improvements in our understanding of the biology of cancers are bringing about a revolution in the development of ‘combined modality therapy’ for patients with apparently locoregionally-confined malignant disease. Numerous studies have shown that platinum-based chemotherapy improves local disease control and often survival in a wide range of tumours treated with radiotherapy, including lung [10], head and neck , rectum and cervix. The combination of accurately delivered radiotherapy with new molecularly-targeted therapies has great therapeutic potential. Tirapazemine is a cytotoxic agent specific for hypoxic cells that shows great promise in combination with radiotherapy [11]. It is now possible to attack specific molecular targets in selected cancers, such as gastrointestinal stromal tumours (GISTs) using imatinib [12], a molecule that specifically targets the surface tyrosine kinase receptor c-Kit (CD117), now recognised as the hallmark immunohistochemical cell marker of GIST. A monoclonal antibody directed at the epidermal growth factor receptor (EGFR), cetuximab [13], has been approved by the U.S. Food and Drugs Administration for the treatment of patients with colorectal cancer who no longer respond to standard chemotherapy treatment with irinotecan. Inhibition of EGFR in combination with radiotherapy may have therapeutic potential in a range of cancers characterised by EGFR overexpression.

In conclusion, these are exciting times in radiotherapy. Advances in radiotherapy technology, and anatomic and functional imaging together with new insights into tumour biology and new pharmaceuticals are leading to rapid developments in our approach to patients with potentially curable cancers. For the foreseeable future, radiotherapy will remain a critically useful tool in our struggle to control malignant disease.
